# The History and Capabilities of Herbal Simples

**Published:** 1893-07-22

**Authors:** W. T. Fernie


### THE HISTORY AND CAPABILITIES OF HERBAL SIMPLES.

By W. T. Fernie, M.D.

### GARLIC AND THE ONION.

(*Cotinnued from page 372, Vol. XII.*)

Garlic has all the qualities of the onion in a more intense form. It consists of several combined small bulbs commonly called cloves, and bears the title of Allium sativum, being named from "gar," a spear, and "leac," plant, because of its sharp, tapering leaves. Also it is popularly called "churl's treacle," from its being regarded as a popular theriacon or antidote to the bite of a viper. Thus we read that juice of garlick "fordop venym and poyson mightily, and is called homly folky's triacle." It grows naturally in Sicily, and was first cultivated in English gardens about 1540, and is the most acrimonious in smell of all the Alliaceous tribe. It was a prime specific for leprosy when that disease prevailed formerly in England, and as the lepers had to pil or peel their own garlic, they were nicknamed "Pil-garlics," whence it came about that anyone who was shunned like a leper had the same epithet applied to him. Stow says, about a person growing old, "He will soon be a peeled garlick like myself." This plant formed a principal ingredient in the "Four Thieves' Vinegar," employed successfully at Marseilles for protection from the plague when prevailing there. Its active properties depend on an essential oil, which is readily obtainable by distillation, the odour of the bulb being very penetrating and diffusible. A medicinal tincture is made from garlic which is stimulating, antispasmodic, expectorant, and diuretic, proving useful in whooping cough, asthma, and other kindred pulmonary affections. The juice may be profitably rubbed into the spines of children until the skin surface becomes red and glowing. Dr. Bowles, a famous English physician, employed garlic as a secret remedy in asthma with considerable success. He made a preserve of the cloves by first boiling them in a closed vessel until quite tender, then drying them carefully with a napkin. To the water in which they had been boiled an equal quantity of the strongest vinegar was added, and to this as much white sugar as was necessary to make a syrup. The same was then poured over the dry bulbs, put in the earthen jar, and carefully stopped for use. One or two of the bulbs were taken by the sufferer each morning while fasting, together with one or two tablespoonfuls of the syrup. The strong, penetrating taste and odour of garlic are offensive to most English palates, but are much relished by the Russians, Poles, and Spaniards, and especially by the Jews. Though the Egyptians and the Romans esteemed the onion and the leek as divinities, yet garlic was detested by the Greeks. It is true the Attic husbandmen had been in the habit of eating it from remote times, probably because the strong odour was useful in driving away venomous creatures. But those persons who partook of garlic were never permitted to enter the Temple of Cybele. Horace was made ill by eating some garlic at a supper with Mœcenas, and he afterwards reviled the plant (in his third epode) as "Cicutis allium nocentius, O, dura messorum ilia!"—"Garlic more baneful than hemlock; how tough must the bowels of the reapers be to digest it!" Or, as spiritedly rendered by Sir Theodore Martin:—

"If his old father's throat any impious sinner
Has cut with unnatural hand to the bone,
Give him garlic, more noxious than hemlock, at dinner!
Ye gods! what strong stomachs the reapers must own!"

*(To be concluded.)*

